# Healthcare resource utilization and costs associated with inflammatory bowel disease among patients with chronic inflammatory diseases: a retrospective cohort study

**DOI:** 10.1186/s41927-020-0115-2

**Published:** 2020-04-02

**Authors:** David P. Hudesman, Soumya D. Chakravarty, Bruno Emond, Lorie A. Ellis, Patrick Lefebvre, Kay Sadik, Jose U. Scher

**Affiliations:** 1 NYU Langone Health, 240 East 38th Street, 23rd Floor, New York, NY 10016 USA; 2 Janssen Scientific Affairs, LLC, 800 Ridgeview Drive, Horsham, PA 19044 USA; 3 Drexel University College of Medicine, Philadelphia, PA USA; 4 Analysis Group, Inc., Montréal, Québec Canada

**Keywords:** Inflammatory bowel disease, Crohn’s disease, Ulcerative colitis, Psoriasis, Psoriatic arthritis, Ankylosing spondylitis, Rheumatoid arthritis, Healthcare costs, Healthcare resource utilization

## Abstract

**Background:**

Chronic inflammatory diseases (CIDs; ankylosing spondylitis [AS], psoriatic arthritis [PsA], psoriasis [PsO], or rheumatoid arthritis [RA]) and inflammatory bowel disease (IBD; Crohn’s disease and ulcerative colitis) are associated with substantial economic burden. The relative increased costs among patients with CIDs and concomitant IBD compared to those without IBD is an important consideration when deciding on the clinical management of patient symptoms. Given the increasing use of novel agents for the treatment of CIDs, including those that may increase the risk of IBD in patients with CIDs, the objective of the study was to describe the incidence of IBD and to quantify healthcare resource utilization (HRU) and costs associated with IBD among patients with CIDs.

**Methods:**

The IBM MarketScan® Research Databases (1/2010–7/2017) were used to identify adult patients with ≥2 claims with a diagnosis of either AS/PsA/PsO/RA (index date was a random claim for AS/PsA/PsO/RA). The one-year incidence rate of IBD was calculated following the index date. HRU and healthcare costs were compared between patients developing and not developing IBD in the year following the index date, adjusting for baseline characteristics.

**Results:**

A total of 537,450 patients with CIDs (mean age = 54.0 years; 63.1% female) were included in the study. The 1-year incidence rate of IBD was 0.52% (range = 0.39% in patients with PsO but without PsA to 1.73% in patients with AS). Patients who developed IBD (*N* = 2778) had significantly higher rates of inpatient, outpatient, and emergency room visits (incidence rate ratios [IRR] = 2.91, 1.35, 1.81; all *P* < 0.0001), compared to patients without IBD (*N* = 534,672). Patients who developed IBD had $18,500 (*P* < 0.0001) higher total costs per year, including $15,121 (*P* < 0.0001) higher medical costs and $3380 higher pharmacy costs (*P* < 0.0001).

**Conclusion:**

Higher HRU and costs were observed in patients with concomitant CID and IBD compared to patients with CID alone. Consideration should be given to treatment decisions that adequately manage CID and IBD to ensure optimal clinical and economic outcomes.

## Background

Inflammatory bowel disease (IBD; Crohn’s disease and ulcerative colitis) is characterized by chronic inflammation of the gastrointestinal tract and is a common extra-articular manifestation in patients with chronic inflammatory diseases (CIDs), such as ankylosing spondylitis (AS), psoriatic arthritis (PsA), psoriasis (PsO), and to a lesser extent in patients with rheumatoid arthritis (RA) [1–4]. Distinct CIDs and IBD share similar genetic susceptibility loci and pathological mechanisms including multiple inflammatory cytokines and immune-signaling pathways [5–10].

Given the pathophysiological relationships between CID and IBD, many pharmacological treatments target manifestations of both syndromes [11–13]. These include disease-modifying anti-rheumatic drugs (DMARDs), corticosteroids, and biologic therapies. However, there is evidence suggesting that some of these treatments may increase the risk of developing new onset IBD or exacerbating existing IBD in patients with CIDs [12, 14, 15]. The use of certain biologic therapies such as etanercept (a tumor necrosis factor inhibitor) and interleukin-17 (IL-17) antagonists in patients with CIDs is cautioned due to possible increased risk of IBD [16–22].

While multiple previous studies have assessed the incremental burden of IBD relative to controls without IBD [23–27], little is known on the burden of IBD in patients with pre-existing CID. A recent real-world analysis of the economic burden associated with IBD among patients with PsA and AS in the United States (US) showed that compared to patients without IBD, those with IBD had significantly higher total healthcare costs-- up to 27% higher in patients with PsA and 38% higher among patients with AS [28]. However, this analysis exclusively focused on patients with PsA or AS; thus, it is unclear whether the conclusions of this study can be generalized to other patients with CID.

The objectives of the current study were to describe the incidence of IBD among patients with CIDs and to compare HRU and costs among patients with CIDs who developed IBD versus patients with CIDs who did not develop IBD.

## Methods

### Data source

This analysis utilized data from the IBM MarketScan® Research Databases (01/01/2010–07/31/2017), which include the Commercial Claims and Encounters database and the Medicare Supplemental and Coordination of Benefits database. The databases comprise claims from approximately 75 million individuals covered by 100 payers. The databases cover all census regions of the US and provide eligibility-related information as well as outpatient medical claims, inpatient medical claims, and outpatient drug dispensing claims information. Data were de-identified and the databases were fully compliant with the Health Insurance Portability and Accountability Act and thus, no ethics board review was required.

### Study design

**Fig. 1:**
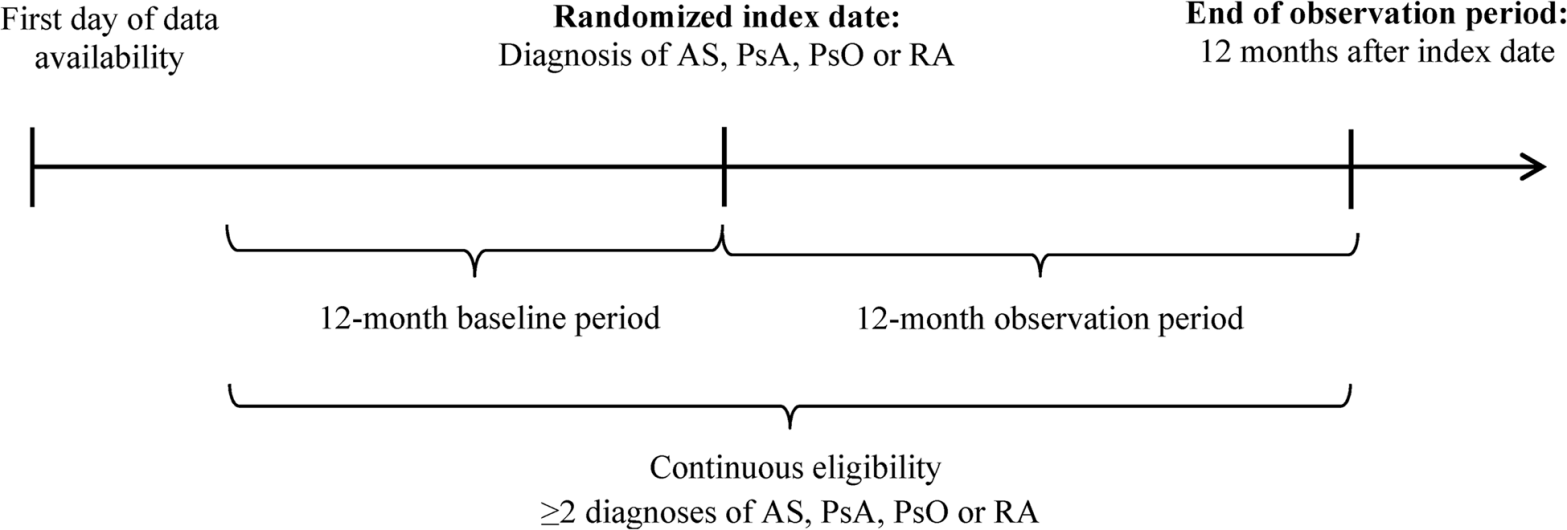
Study design scheme. RA, rheumatoid arthritis; PsA, psoriatic arthritis; PsO, psoriasis; AS, ankylosing spondylitis

A retrospective longitudinal cohort study design was used. The index date for patients with AS, PsA, PsO, or RA was randomly assigned among service dates with a claim related to these CIDs. The rationale was to capture a prevalent population of patients with CIDs and to minimize the potential bias associated with the selection of the most or least recent dates associated with such claims, which could lead to the inclusion of patients with new-onset or advanced CID. When evaluating the incidence of IBD, a non-CID population was also studied to put in perspective the incidence of IBD in patients with CIDs to that of the general population. For the non-CID population, the index date was randomly assigned among all dates for which a service was provided. The baseline period was defined as the 12-month period prior to the index date. The observation period was defined as the 12-month period following the index date (i.e., patients were censored 12 months after the index date; Fig. 1).

### Study sample

To be included in the study sample, patients were required to be ≥18 years of age as of the index date and have ≥12 months of continuous health plan enrollment pre- and post-index date. Patients in the CID cohort were required to have ≥2 claims with the same diagnosis among the following CIDs: AS (International Classification of Diseases, Ninth Revision, Clinical Modification [ICD-9 CM] code 720.0; International Classification of Diseases, Tenth Revision, Clinical Modification [ICD-10 CM] code M45.x), PsA (ICD-9 CM code 696.0; ICD-10 CM code L40.5x), PsO (ICD-9 CM code 696.1; ICD-10 CM codes L40.0-L40.4, L40.8, or L40.9), or RA (ICD-9 CM code 714.0; ICD-10 CM codes M05.1-M05.9, or M06). Patients with ≥2 claims with a diagnosis of IBD (i.e., Crohn’s disease [ICD-9 CM code 555.x; ICD-10 CM code K50.x] or ulcerative colitis [ICD-9 CM code 556.x; ICD-10 CM code K51.x]) during the 12-month baseline period were excluded. Moreover, patients with cancer treated with rituximab or ofatumumab during the baseline period were excluded because it could not be determined using claims data whether these agents were prescribed to treat cancer or CID. Patients with a transplant procedure were also excluded because of the high costs usually associated with this procedure, which may skew results. The non-CID cohort was composed of individuals with no claim with a diagnosis of AS, PsA, PsO, or RA at any time. Similar to the CID cohort, patients in the non-CID cohort were additionally required to be ≥18 years of age as of the index date and have ≥12 months of continuous health plan enrollment pre- and post-index date. Patients with cancer treated with rituximab or ofatumumab, with a transplant procedure, or with ≥2 claims with a diagnosis of IBD during the 12-month baseline period were excluded.

### Study cohorts

Patients with CIDs were classified into six cohorts: (1) all patients with CIDs (i.e., AS, PsA, PsO, or RA); (2) patients with RA; (3) patients with PsO and PsA; (4) patients with PsO but without PsA; (5) patients with AS; and (6) patients with AS, PsA, or PsO. Eligible patients with claims for more than one CID were classified into each eligible CID cohort (i.e., one patient could be present in more than one cohort). Each of the six cohorts mentioned above was analyzed separately.

### Study measurements

Baseline characteristics included age, gender, type of insurance plan, region of residence, year of index date, Quan-Charlson Comorbidity Index (Quan-CCI), extra-articular manifestations (EAMs), gastro-related conditions, drug use, all-cause HRU, and all-cause costs.

Patients developing IBD were identified by the presence of ≥2 claims with a diagnosis of IBD during the course of the observation period. The one-year incidence rate (IR) of IBD was calculated for each cohort. The numerator was defined as the number of patients with ≥2 claims with an IBD diagnosis during the period of evaluation (i.e., 1 year), and the denominator was defined as the total number of patients in the cohort studied. As a sensitivity analysis, the one-year prevalence of IBD among patients with or without a prior IBD diagnosis was also reported for each cohort. The numerator and denominator definitions used were the same as the ones used to measure incidence, except that patients who had a prior IBD diagnosis were not excluded from the numerator and denominator.

HRU outcomes included number of inpatient admissions, number of days of inpatient stay, number of days with outpatient services, number of days with emergency room (ER) visits, and number of days with durable medical equipment use. The number of days with a surgery was also evaluated.

All-cause total healthcare costs were stratified by medical and pharmacy costs. Medical costs were further stratified by inpatient, outpatient, ER, and durable medical equipment costs. Costs related to surgery were also evaluated. By definition, all-cause healthcare costs included any healthcare costs incurred during the follow-up period, regardless of whether they were related to CIDs, IBD (e.g., endoscopies, colonoscopies), or other comorbidities.

### Statistical analysis

Baseline characteristics were summarized using means and standard deviations (SDs) for continuous variables and proportions for categorical variables. The one-year IR of IBD was reported as a proportion of patients.

The rates of HRU between patients with and without IBD among the six cohorts were compared using generalized linear models (GLMs) with a Poisson distribution adjusting for age, gender, region, type of insurance plan, Quan-CCI, and type of CID (i.e., RA, PsA, PsO, or AS); incidence rate ratios (IRRs) with corresponding 95% confidence intervals (CIs) and *p*-values were reported. GLMs with normal distribution were used to compare costs between patients with and without IBD among the six cohorts, adjusting for age, gender, region, type of insurance plan, Quan-CCI score (a score derived from a number of comorbidities [e.g., hypertension, diabetes, chronic pulmonary disease] and their associated risk of in-hospital mortality [29]), and type of CID (i.e., AS, PsA, PsO, or AS); mean yearly cost differences (MYCDs) and the corresponding 95% CIs and *p*-values were reported. Non-parametric bootstrap procedures were used to evaluate statistical significance and 95% CIs. In a sensitivity analysis, costs were evaluated in the subset of patients with AS, RA, PsA, or PsO who received DMARDs and/or corticosteroids.

Cost and HRU outcomes were evaluated per-patient-per-year (PPPY). Cost outcomes were inflated to 2017 US dollars (USD) using the medical component of the US Consumer Price Index.

## Results

### Baseline characteristics

**Fig. 2:**
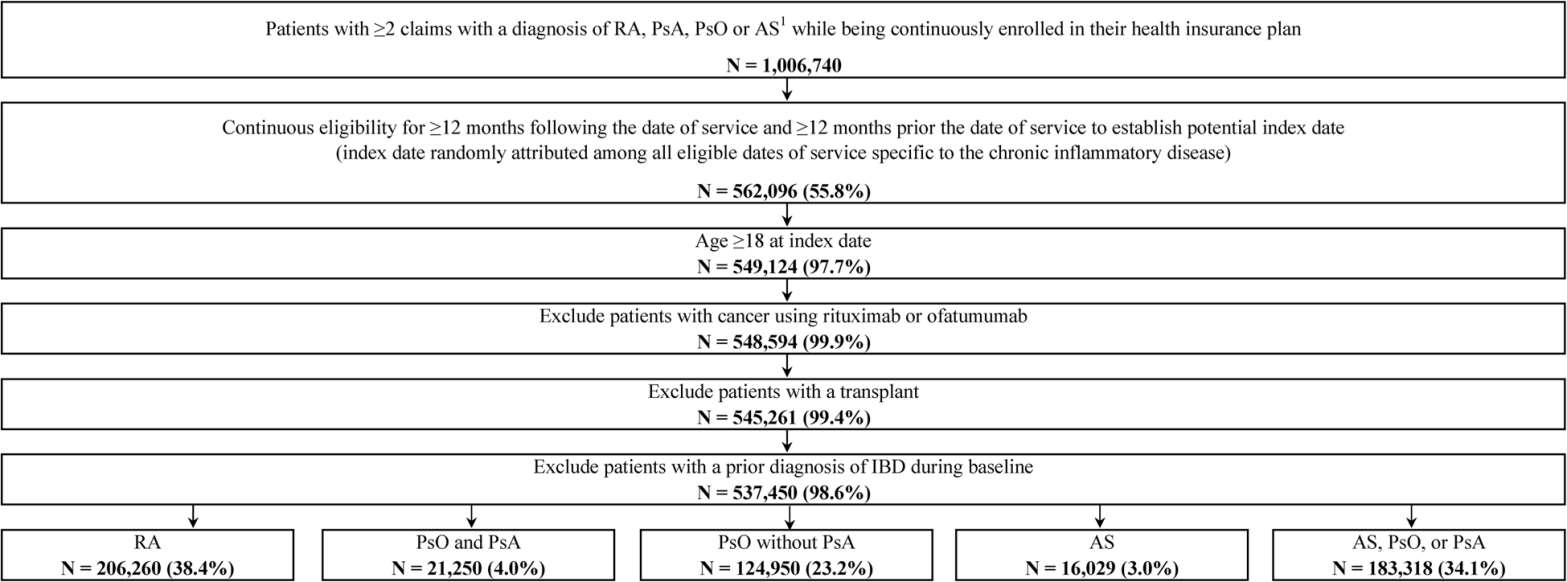
Identification of the study population. RA, rheumatoid arthritis; PsA, psoriatic arthritis; PsO, psoriasis; AS, ankylosing spondylitis; IBD, inflammatory bowel disease. Notes: 1. Identified using the following ICD-9-CM and ICD-10-CM codes: RA (ICD-9-CM code 714.0; ICD-10-CM code: M05.1-M05.9, M06), PsA (ICD-9-CM code 696.0; ICD-10-CM code: L40.5x), PsO (ICD-9-CM code 696.1; ICD-10-CM code: L40.0-L40.4, L40.8, L40.9), or AS (ICD-9-CM code 720.0; ICD-10-CM code: M45.x)

A total of 537,450 patients with CIDs, including 206,260 patients with RA; 21,250 patients with PsO and PsA; 124,950 patients with PsO but without PsA; 16,029 patients with AS; and 183,318 with AS, PsO, or PsA composed the study’s analytical sample (Fig. 2). Between the RA, PsO with PsA, PsO without PsA, and AS patient cohorts, 3280 patients were both in the RA and PsO with PsA cohorts, 3310 patients were both in the RA and PsO without PsA cohorts, 2957 patients were both in the RA and AS cohorts, 276 patients were both in the PsO with PsA and AS cohorts, and 288 patients were both in the PsO without PsA and AS cohorts. A total of 1,008,436 patients were not diagnosed with CIDs.

In the CID and non-CID cohorts, mean age was 54.0 and 46.3 years, respectively; 63.1 and 55.1% were female. In the other condition-specific cohorts, mean age ranged from 49.4 years in the AS cohort to 57.5 years in the RA cohort, and the proportion of female patients ranged from 51.3% in the AS, PsO or PsA cohort to 75.8% in the RA cohort. The most commonly used medications at baseline were corticosteroids (45.3%), opioids (38.8%), DMARDs (37.6%) and non-steroidal anti-inflammatory drugs (NSAIDs; 33.3%). In the non-CID cohort, the most commonly used medications were opioids (24.0%), corticosteroids (19.5%), and NSAIDS (17.0%). A higher proportion of patients in the RA cohort were using corticosteroids and DMARDs (62.3 and 67.1%, respectively), and a higher proportion of patients in the PsO and PsA cohort were using biologics (54.3%) compared to other cohorts (Table 1).

**Table 1:** Demographic and clinical characteristics evaluated during the 12-month baseline period

	Non-CID cohort (not diagnosed with RA, PsA, PsO or AS)	All patients: RA, PsO, PsA, or AS cohort	Patients with RA	Patients with PsO and PsA	Patients with PsO but without PsA	Patients with AS	Patients with AS, PsO or PsA
	(*N* = 1,008,436)	(*N* = 537,450)	(*N* = 206,260)	(*N* = 21,250)	(*N* = 124,950)	(*N* = 16,029)	(*N* = 183,318)
Age^a^, mean ± SD	46.3 ± 16.3	54.0 ± 14.7	57.5 ± 13.7	51.4 ± 12.2	51.1 ± 15.1	49.4 ± 13.8	51.3 ± 14.4
Female, %	55.1%	63.1%	75.8%	52.8%	51.5%	43.8%	51.3%
Payment type, %							
Commercial	88.9%	79.3%	73.6%	89.0%	83.6%	88.3%	84.9%
Medicare	11.1%	20.7%	26.4%	11.0%	16.4%	11.7%	15.1%
Region of residence^a^, %							
South	39.0%	37.5%	39.1%	40.2%	36.2%	36.3%	36.8%
North Central	21.9%	22.4%	23.0%	20.2%	22.4%	19.1%	21.7%
West	18.9%	16.8%	16.5%	17.6%	16.7%	24.9%	17.4%
North East	18.9%	22.4%	20.5%	21.0%	24.0%	18.9%	23.2%
Unknown	1.4%	0.8%	0.8%	1.1%	0.8%	0.8%	0.9%
Year of index date, %							
2011	30.9%	24.8%	28.2%	23.4%	26.0%	25.7%	25.9%
2012	19.2%	17.8%	17.5%	14.4%	17.8%	16.6%	17.4%
2013	16.8%	15.8%	15.1%	14.6%	15.6%	14.8%	15.4%
2014	13.0%	13.4%	12.3%	13.4%	13.5%	12.5%	13.3%
2015	16.9%	16.0%	14.5%	19.1%	15.8%	16.9%	16.2%
2016	3.3%	12.2%	12.4%	15.1%	11.3%	13.5%	11.9%
Quan-Charlson Comorbidity Index^b^, mean ± SD	0.5 ± 1.1	1.2 ± 1.5	1.9 ± 1.4	0.9 ± 1.3	0.7 ± 1.2	0.9 ± 1.3	0.7 ± 1.3
Extra-articular manifestations^b,c^, %	12.4%	43.5%	27.3%	100.0%	100.0%	32.5%	84.8%
Gastro-related conditions^b,d^, %	13.6%	18.8%	22.1%	19.5%	15.3%	22.5%	16.7%
Drug use^b^, %							
NSAIDS	17.0%	33.3%	43.7%	42.5%	20.5%	51.5%	28.3%
Corticosteroids	19.5%	45.3%	62.3%	49.3%	33.4%	47.7%	38.2%
Biologics	0.1%	21.0%	31.9%	54.3%	16.4%	44.9%	27.0%
PDE4 inhibitors	0.0%	0.4%	0.1%	3.2%	0.9%	0.1%	1.2%
Opioids	24.0%	38.8%	50.0%	42.3%	27.6%	50.0%	33.1%
DMARDs	1.5%	37.6%	67.1%	56.4%	17.4%	39.4%	28.8%

*CID* Chronic inflammatory disease, *SD* Standard deviation, *HRU* Healthcare resource utilization, *RA* Rheumatoid arthritis, *PsA* Psoriatic arthritis, *PsO* Psoriasis, *AS* Ankylosing spondylitis, *NSAIDs* Non-steroidal anti-inflammatory drugs, *DMARDS* Disease modifying anti-rheumatic drugs

^a^Measured at the index date.

^b^Measured during the 12-month baseline period.

^c^Extra-articular manifestations include cutaneous, ocular, cardiovascular, urogenital, pulmonary, and other manifestations such as enthesopathies, parapsoriasis, pityriasis, and other psoriasis and similar disorders.

^d^Gastro-related conditions include diarrhea, weight loss, blood in stool, abdominal pain, gastrointestinal hemorrhage, ischemic colitis, dyspepsia, and gastroenteritis.

### One-year IR and prevalence of IBD

**Fig. 3:**
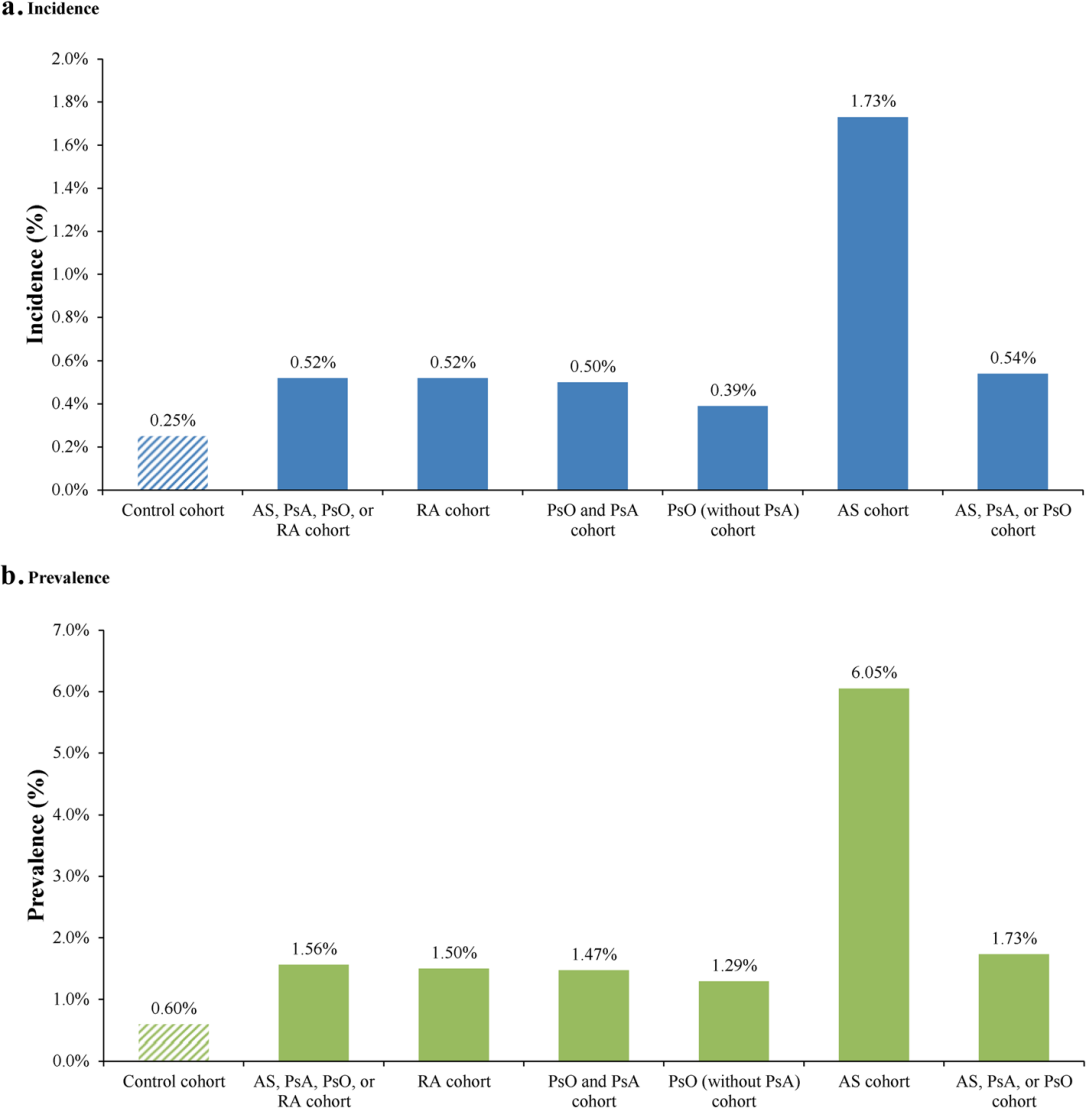
One-year incidence rate (**a**) and prevalence (**b**) of IBD by type of CID. CID, chronic inflammatory disease; RA, rheumatoid arthritis; PsA, psoriatic arthritis; PsO, psoriasis; AS, ankylosing spondylitis; IBD, inflammatory bowel disease

The one-year IR and prevalence of IBD by type of CID are depicted in Fig. 3a and Fig. 3b, respectively. Among all patients with CIDs, the IR of IBD was 0.52%. For patients in the AS cohort, the IR of IBD was numerically higher (1.73%), compared to other CID cohorts (IR range: 0.39% in the PsO without PsA cohort to 0.54% in the AS, PsA, or PsO cohort) and the non-CID cohort (0.25%). The prevalence of IBD was higher across all cohorts, but consistent trends were observed, with values ranging from 1.29% in patients with PsO without PsA to 6.05% in patients with AS. The prevalence of IBD was 0.60% in the non-CID cohort.

### HRU

**Fig. 4:**
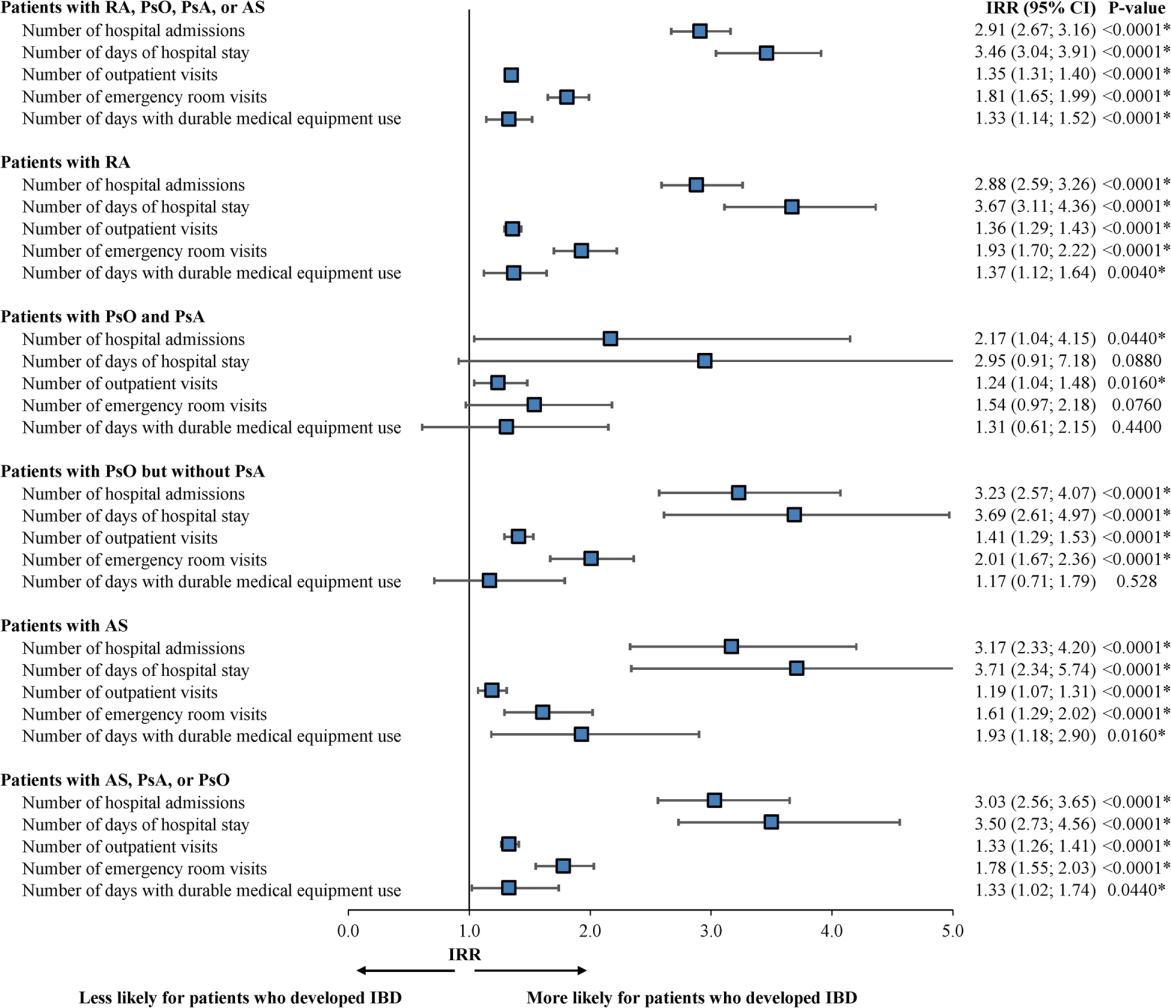
Comparison of HRU during the 12-month observation period between patients developing versus not developing IBD. RA, rheumatoid arthritis; PsA, psoriatic arthritis; PsO, psoriasis; AS, ankylosing spondylitis; IBD, inflammatory bowel disease; HRU, healthcare resource utilization; IRR, incidence rate ratio; CI, confidence interval. * indicates that *p*-value < 0.05

Among patients with CIDs, patients who developed IBD had higher rates of hospital admissions (IRR = 2.91, 95% CI = 2.67–3.16, *P* < 0.0001), days spent at the hospital (IRR = 3.46, 95% CI = 3.04–3.91, *P* < 0.0001), outpatient visits (IRR = 1.35, 95% CI = 1.31–1.40, *P* < 0.0001), and ER visits (IRR = 1.81, 95% CI = 1.65–1.99, *P* < 0.0001) compared to those who did not develop IBD. Similarly, among condition-specific cohorts, those who developed IBD had higher rates of hospital admissions, days spent at the hospital, outpatient visits, and ER visits compared to patients who did not develop IBD (Fig. 4). In addition, among patients with CIDs, compared to those who did not develop IBD, patients who developed IBD were more likely to have an IBD-related surgery (odds ratio = 11.77 95% CI = 9.93–13.96), such as bowel resection or pouch surgery/proctocolectomy with ileal pouch-anal anastomosis.

### Healthcare costs

Among patients with CIDs, unadjusted mean all-cause total healthcare costs per year among those who developed IBD were $41,150 versus $20,644 among those who did not develop IBD. Similar trends were observed in condition-specific cohorts. In these cohorts, total costs ranged from $35,062 (patients with PsO but without PsA) to $50,861 (patients with RA) for patients with IBD and from $16,494 (patients with PsO but without PsA) to $32,452 (patients with PsO and PsA) for patients without IBD. The largest proportion of healthcare costs were attributed to medical costs (Table 2).

**Table 2:** Unadjusted costs by type of chronic inflammatory disease during the 12-month observation period

	Unadjusted all-cause costs per year, USD (2017), mean ± SD [median]	
	Patients who developed IBD	Patients who did not develop IBD
**All Patients: Patients with RA, PsO, PsA, or AS**	***N* = 2778**	***N* = 534,672**
Total healthcare costs	41,150 ± 62,218 [23,482]	20,644 ± 39,510 [7867]
Medical costs	29,735 ± 59,281 [10,781]	13,004 ± 35,906 [3051]
Hospitalization costs	13,163 ± 48,359 [0]	4059 ± 23,848 [0]
Outpatient costs	14,595 ± 25,168 [6399]	8063 ± 21,660 [2426]
Emergency room costs	1836 ± 5954 [0]	771 ± 3706 [0]
Durable medical equipment costs	142 ± 716 [0]	112 ± 762 [0]
Pharmacy costs	11,414 ± 18,916 [3917]	7640 ± 15,353 [1462]
**Patients with RA**	***N* = 1082**	***N* = 205,178**
Total healthcare costs	50,861 ± 71,823 [32,696]	27,026 ± 43,583 [14,242]
Medical costs	38,597 ± 69,336 [15,748]	17,820 ± 40,349 [4836]
Hospitalization costs	17,888 ± 58,399 [0]	5530 ± 26,284 [0]
Outpatient costs	18,319 ± 26,492 [8400]	11,195 ± 24,592 [3651]
Emergency room costs	2200 ± 7188 [0]	950 ± 4371 [0]
Durable medical equipment costs	189 ± 894 [0]	144 ± 825 [0]
Pharmacy costs	12,264 ± 20,941 [4715]	9206 ± 16,335 [2184]
**Patients with PsO and PsA**	***N* = 106**	***N* = 21,144**
Total healthcare costs	50,162 ± 57,420 [35,973]	32,452 ± 38,332 [24,341]
Medical costs	30,747 ± 54,422 [11,781]	14,702 ± 32,814 [3508]
Hospitalization costs	9988 ± 42,153 [0]	3607 ± 19,405 [0]
Outpatient costs	19,042 ± 25,040 [7382]	10,236 ± 22,143 [2843]
Emergency room costs	1581 ± 4740 [0]	755 ± 3772 [0]
Durable medical equipment costs	136 ± 517 [0]	104 ± 763 [0]
Pharmacy costs	19,415 ± 22,960 [10,857]	17,750 ± 21,576 [8425]
**Patients with PsO but without PsA**	***N* = 490**	***N* = 124,460**
Total healthcare costs	35,062 ± 56,249 [17,763]	16,494 ± 36,155 [5807]
Medical costs	24,355 ± 52,492 [8468]	8902 ± 32,175 [2067]
Hospitalization costs	10,940 ± 42,213 [0]	2746 ± 22,967 [0]
Outpatient costs	11,646 ± 23,071 [5330]	5498 ± 18,670 [1681]
Emergency room costs	1665 ± 4635 [0]	568 ± 2858 [0]
Durable medical equipment costs	104 ± 564 [0]	89 ± 641 [0]
Pharmacy costs	10,708 ± 16,664 [3533]	7592 ± 15,405 [1326]
**Patients with AS**	***N* = 277**	***N* = 15,752**
Total healthcare costs	47,410 ± 53,738 [34,535]	28,325 ± 41,809 [17,443]
Medical costs	31,894 ± 53,244 [12,097]	16,045 ± 37,433 [3961]
Hospitalization costs	12,165 ± 42,744 [0]	4242 ± 22,895 [0]
Outpatient costs	17,493 ± 27,693 [6757]	10,861 ± 24,478 [3162]
Emergency room costs	2089 ± 6233 [0]	819 ± 3427 [0]
Durable medical equipment costs	147 ± 661 [0]	123 ± 944 [0]
Pharmacy costs	15,516 ± 18,356 [9058]	12,280 ± 19,354 [3021]
**Patients with AS, PsA, or PsO**	***N* = 982**	***N* = 182,336**
Total healthcare costs	42,074 ± 60,394 [25,759]	20,734 ± 37,661 [8332]
Medical costs	28,425 ± 57,753 [10,267]	10,858 ± 33,031 [2443]
Hospitalization costs	11,719 ± 47,236 [0]	3114 ± 22,291 [0]
Outpatient costs	14,843 ± 25,322 [6313]	7021 ± 20,265 [1998]
Emergency room costs	1747 ± 4986 [0]	628 ± 3091 [0]
Durable medical equipment costs	116 ± 565 [0]	95 ± 684 [0]
Pharmacy costs	13,649 ± 18,296 [5311]	9877 ± 17,339 [1846]

*RA* Rheumatoid arthritis, *PsA* Psoriatic arthritis, *PsO* Psoriasis, *AS* Ankylosing spondylitis, *IBD* Inflammatory bowel disease, *USD* US dollars

**Fig. 5:**
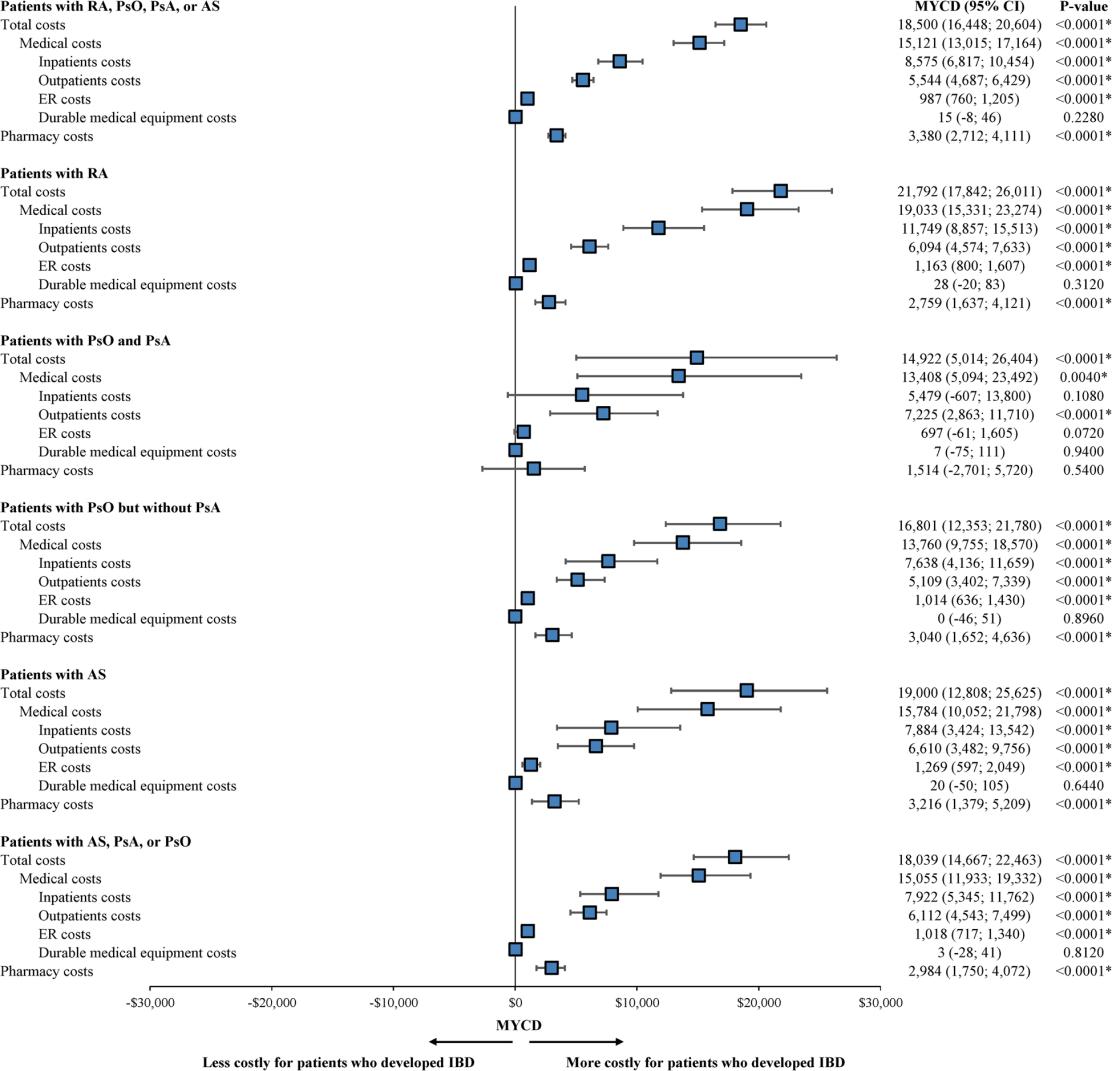
Comparison of costs during the 12-month observation period between patients developing versus not developing IBD. RA, rheumatoid arthritis; PsA, psoriatic arthritis; PsO, psoriasis; AS, ankylosing spondylitis; IBD, inflammatory bowel disease; MYCD, mean yearly cost difference; CI, confidence interval. * indicates that *p*-value < 0.05

Compared to those who did not develop IBD, patients with CID who developed IBD had an average of $18,500 (95% CI = $16,448–$20,604; *P* < 0.0001) higher total costs per year, including $15,121 (95% CI = $13,015–$17,164; *P* < 0.0001) higher medical costs and $3380 (95% CI = $2712–$4111; *P* < 0.0001) higher pharmacy costs. The higher medical costs were driven by higher hospitalization costs (adjusted MYCD: $8575, *P* < 0.0001) and outpatient costs (adjusted MYCD: $5544, *P* < 0.0001). The largest components of pharmacy cost among all CID patients were biologics ($6706 versus $4769; adjusted MYCD: $1771, *P* < 0.0001) and DMARDs ($4917 versus $2047; adjusted MYCD: $2751, *P* < 0.0001). Similar cost differences were observed among condition-specific cohorts. The total annual cost difference between CID with IBD and CID alone ranged from $14,922 in the PsO and PsA cohort, to $21,792 in the RA cohort; differences in medical costs ranged from $13,408 in the PsO and PsA cohort to $19,033 in the RA cohort, and differences in pharmacy costs ranged from $1514 in the PsO and PsA cohort to $3216 in the AS cohort (Fig. 5).

Compared to those who did not develop IBD, patients with CID who developed IBD incurred higher IBD surgery-related costs ($1038 versus $73; adjusted MYCD: $959, *P* < 0.0001). Among the subset of CID patients who underwent IBD surgery, the cost of such a surgery was >$18,000.

Among patients with CIDs who received DMARDs and/or corticosteroids, those who developed IBD (*N* = 2020) incurred mean total all-cause healthcare costs of $44,192 compared to $25,472 for those who did not (*N* = 320,561), resulting in an adjusted MYCD of $17,605 (*P* < 0.0001). Similar to the main analysis, this difference was largely driven by higher hospitalization costs (adjusted MYCD: $7755), outpatient costs (adjusted MYCD: $5392), and pharmacy costs (adjusted MYCD: $3612, all *P* < 0.0001).

## Discussion

This study is among the first to provide estimates of real-world incidence and economic burden of IBD among patients with CIDs, overall, and separately in subsets of patients with RA, patients with PsO and PsA, patients with PsO but without PsA, patients with AS, and patients with AS, PsA, or PsO. Overall, the incidence of IBD among patients with CIDs was 0.52% and was 0.25% in the non-CID cohort. Patients with CIDs who developed IBD incurred on average $14,922–$21,792 higher total healthcare costs and had higher HRU per year, including hospital admissions, days spent at the hospital, outpatient visits, and ER visits compared to patients without IBD. Important drivers of the cost difference included hospitalization costs, outpatient costs, and costs related to the use of biologics and DMARDs. Another possible driver could be costs related to endoscopy/colonoscopy procedures, which were captured as part of all-cause healthcare costs, but were not reported separately.

The one-year incidence of IBD varied among subsets of patients with different CIDs, ranging from 0.39% in patients with PsO but without PsA to 1.73% among patients with AS. The one-year prevalence of IBD was higher across all cohorts, but consistent trends were observed, with values ranging from 1.29% in patients with PsO but without PsA to 6.05% in patients with AS. A previous study based on similar data reported IBD incidence of 2.5% in patients with PsA and 4.1% in patients with AS during the one-year period following the initial claim for PsA/AS [28]. IRs were higher because the definition of IBD included Crohn’s disease, ulcerative colitis, and gastrointestinal disturbances (i.e., gastroenteritis, colitis, and gastritis) as opposed to only Crohn’s disease and ulcerative colitis in the current study. Although crude IRs vary between previous estimates and the present study, both suggest a higher rate of IBD among patients with AS compared to other CID types. In a systematic review of 156 studies, the pooled prevalence of IBD among patients with AS was 6.8% [30], consistent with the one-year prevalence of IBD among patients with AS reported in the current study (6.05%). In addition, the IR of IBD in patients with PsO and PsA was higher than in patients with PsO without PsA. This result is consistent with the literature, as a previous study showed that patients with concomitant PsO and PsA were almost twice as likely to develop IBD as patients with PsO only [31]. Having PsA in addition to PsO may also explain why the observed IR of IBD was more similar between the RA cohort and the PsO with PsA cohort than between the RA cohort and the PsO without PsA cohort.

While it was anticipated that patients with IBD and CID would incur higher costs than patients with CID alone, this study is the first to quantify the incremental costs and HRU associated with IBD among patients with AS, PsA, PsO, or RA, stratified by type of CID. Of note, the difference in total all-cause healthcare costs found in our study ($18,500) is similar to that observed in recent analyses which assessed the incremental burden of patients with IBD versus non-IBD controls (IBD: $16,031 [27], CD only: $17,463 [32], UC only: $11,029 [33]). Although comparisons with results from other analyses are prone to confounding, this suggests that the incremental burden associated with IBD in a population of patients with CID is similar to that observed in the general population.

Only one previous analysis from Bergman et al. compared HRU and costs between patients with CID who subsequently developed IBD and controls who did not develop IBD [28]. However, this study only included patients with PsA or AS, thereby precluding the generalization of findings to other CIDs. Indeed, CIDs show substantial heterogeneity in their incidence, prevalence, and the characteristics of affected patients (i.e., age, gender, associated comorbidities, etc.) [34], which may impact HRU and costs. For this reason, all patients with CIDs were included in the present study and were classified into different cohorts based on the type of CID(s) diagnosis.

The total annual direct costs of all patients with IBD in the US in 2014 was estimated to be between $11 billion and $28 billion [35]. With the rising prevalence of both Crohn’s disease and ulcerative colitis, IBD represents the leading chronic gastrointestinal disease with increasing healthcare expenditures in the US [36]. There has been a shift in IBD costs from inpatient to outpatient care since the introduction of biologic therapies as the standard of care [36]. Biologic agents are increasingly used to treat both CID and IBD when conventional therapies fail, such as in CID patients unresponsive or intolerant to NSAIDs [37]. While some biologics are approved for the treatment of IBD (i.e., certolizumab pegol, subcutaneous golimumab, adalimumab, infliximab, vedolizumab, and natalizumab), biologic therapies with certain mechanisms of action, such as IL-17 antagonists and etanercept, have been linked with an increased risk of new onset IBD or exacerbation of existing IBD. A number of studies have noted that patients with Crohn’s disease experienced exacerbation of their symptoms after receiving treatment with secukinumab [14] or brodalumab [15]. Evidence from clinical trials has also shown higher incidence of IBD in patients treated with different IL-17 antagonists [14, 16, 17]. Similarly, a large real-world retrospective longitudinal cohort study of the incidence of IBD among patients with CIDs treated with IL-17 blockers or PDE4 inhibitors found that treatment with IL-17 antagonists was associated with more than 3-fold higher odds of having IBD relative to patients naïve to biologics and patients treated with biologics not indicated for the treatment of IBD [38]. Finally, a number of studies also found that etanercept was associated with exacerbation of existing IBD or new onset IBD [21, 22].

Even if the incidence of IBD remains low (as was shown in the previous studies and by the results of the current study), the occurrence of concomitant IBD in a few cases was shown to be associated with a considerable increase in total healthcare costs for patients with CIDs. It may also result in adverse outcomes [39], especially in patients who already have difficult chronic conditions such as AS, PsA, PsO, or RA. The findings of the present study suggest that treatment decisions should consider the increased risk and associated HRU and healthcare costs of IBD. Healthcare providers should be aware of the cost burden of IBD and treatments that optimize cost-efficiency when treating patients with CID.

The high rates of opioid use observed in the current study reflect both the pain burden associated with CIDs as well as US-specific circumstances. Overall, 38.8% of patients with CIDs and 24.0% of those without CIDs exhibited opioid use at baseline. This difference is likely driven by patients who were prescribed opioids to manage the pain associated with CIDs. Patients with CIDs are known to exhibit higher use of prescription opioids than the general population for pain management [40, 41]. A study by Zhdanava et al. that included patients with PsO reported rates of opioid use that were consistent with those of the present study (PsO cohort: 42.8%, matched non-PsO cohort: 30.7%) [41]. In absolute terms, the rate of opioid use in the non-CID cohort of our study (24.0%) was nonetheless high, and that reported by Zhdanava et al. (30.7%) was in the same range. This is likely due to the marked increase of opioid use across the US over the past few years [42].

The strengths of the study include its large sample size and estimation of incidence, HRU, and healthcare costs and its components in an overall sample of patients with CIDs as well as among subsets of patients with specific CIDs, which has never been published before. The study findings should also be interpreted in the context of its limitations. First, claims databases may contain inaccuracies from missing data and occasional coding errors. Second, as with all observational studies, results from the current study may be affected by residual confounding from measured and unmeasured factors. In particular, since incidence rates of IBD were only reported descriptively for each patient cohort, confounding related to differences in patients’ baseline characteristics (such as age) may remain. Third, the data used in this study covered only commercially-insured patients who may have a higher socioeconomic status and may not be representative of the general US population. For example, uninsured individuals and Medicaid beneficiaries were not included in the current study. Fourth, when describing the one-year IR of IBD, no adjustments for potential confounders were made to account for differences in characteristics between the cohorts; therefore, no statistical comparisons between cohorts were conducted for IRs. Finally, patients may have had PsA for many years. Therefore, the number of patients with PsO and PsA may have been underestimated in the current study among patients with PsA, given that there may have been no claim for PsO among patients with PsA in recent years.

## Conclusions

In patients with CIDs, developing IBD was associated with substantially higher HRU and healthcare costs compared to patients not developing IBD. Treatment decisions for patients with CIDs should factor in the risk of developing of IBD as well. Such considerations have the potential to improve cost-effectiveness and health outcomes for patients with CIDs.

## Data Availability

The data that support the findings of this study are available from IBM but restrictions apply to the availability of these data, which were used pursuant to a data use agreement. The data are available through requests made directly to IBM, subject to IBM’s requirements for data access.

## Abbreviations

AS: Ankylosing spondylitis
CI: Confidence interval
CID: Chronic inflammatory disease
DMARD: Disease-modifying anti-rheumatic drug
EAM: Extra-articular manifestations
ER: Emergency room
GLM: Generalized linear model
HRU: Healthcare resource utilization
IBD: Inflammatory bowel disease
ICD-10 CM: International Classification of Diseases, Tenth Revision, Clinical Modification
ICD-9 CM: International Classification of Diseases, Ninth Revision, Clinical Modification
IL-17: Interleukin-17
IR: Incidence rate
IRR: Incidence rate ratios
MYCD: Mean yearly cost difference
NSAID: Non-steroidal anti-inflammatory drug
PDE4: Phosphodiesterase 4
PPPY: Per-patient-per-year
PsA: Psoriatic arthritis
PsO: Psoriasis
Quan-CCI: Quan-Charlson Comorbidity Index
RA: Rheumatoid arthritis
SD: Standard deviation
US: United States
